# Crystal structure of *catena*-poly[[cadmium(II)-di-μ_2_-bromido-μ_2_-l-proline-κ^2^ *O*:*O*′] monohydrate]

**DOI:** 10.1107/S2056989015001176

**Published:** 2015-01-24

**Authors:** S. Sathiskumar, T. Balakrishnan, K. Ramamurthi, S. Thamotharan

**Affiliations:** a Crystal Growth Laboratory, PG & Research Department of Physics, Periyar EVR College (Autonomous), Tiruchirappalli 620 023, India; b Crystal Growth and Thin Film Laboratory, Department of Physics and Nanotechnology, SRM University, Kattankulathur 603 203, India; c Department of Bioinformatics, School of Chemical and Biotechnology, SASTRA University, Thanjavur 613 401, India

**Keywords:** crystal structure, l-proline cadmium bromide, cadmium coordination polymer, N/O—H⋯Br/O hydrogen bonds, distorted octa­hedral geometry.

## Abstract

In the title coordination polymer, {[CdBr_2_(C_5_H_9_NO_2_)]·H_2_O}_*n*_, the Cd^II^ ion is coordinated by four bromido ligands and two carboxyl­ate oxygen atoms of two symmetry-related proline ligands, which exist in a zwitterionic form, in a distorted octa­hedral geometry. There is an intra­molecular N—H⋯O hydrogen bond between the amino group and the carboxyl­ate fragment. Each coordinating ligand bridges two Cd^II^ atoms, thus forming polymeric chains running along the *c-*axis direction. The water mol­ecules of crystallization serve as donors for the weak inter­molecular O—H⋯O and O—H⋯Br hydrogen bonds that link adjacent polymeric chains, thus forming a three-dimensional structure. N—H⋯O and N—H⋯Br hydrogen bonds also occur.

## Abstract 2

In the title salt, crystalline water mol­ecules serve as donors for the weak inter­molecular O—H⋯O and O—H⋯Br hydrogen bonds which link adjacent polymeric chains.

## Chemical context   

The characterization of second-order non-linear optical (NLO) materials is important because of their potential applications such as frequency shifting, optical modulation, optical switching, telecommunication and signal processing. It is known that the chiral amino acids and their complexes are potential materials for NLO applications (Eimerl *et al.*, 1989 ▸; Pal *et al.*, 2004 ▸; Srinivasan *et al.*, 2006 ▸). This study is a part of an ongoing investigation of the crystal and mol­ecular structures of a series of amino acid–metal complexes (Sathiskumar *et al.*, 2015 ▸; Balakrishnan *et al.*, 2013 ▸).
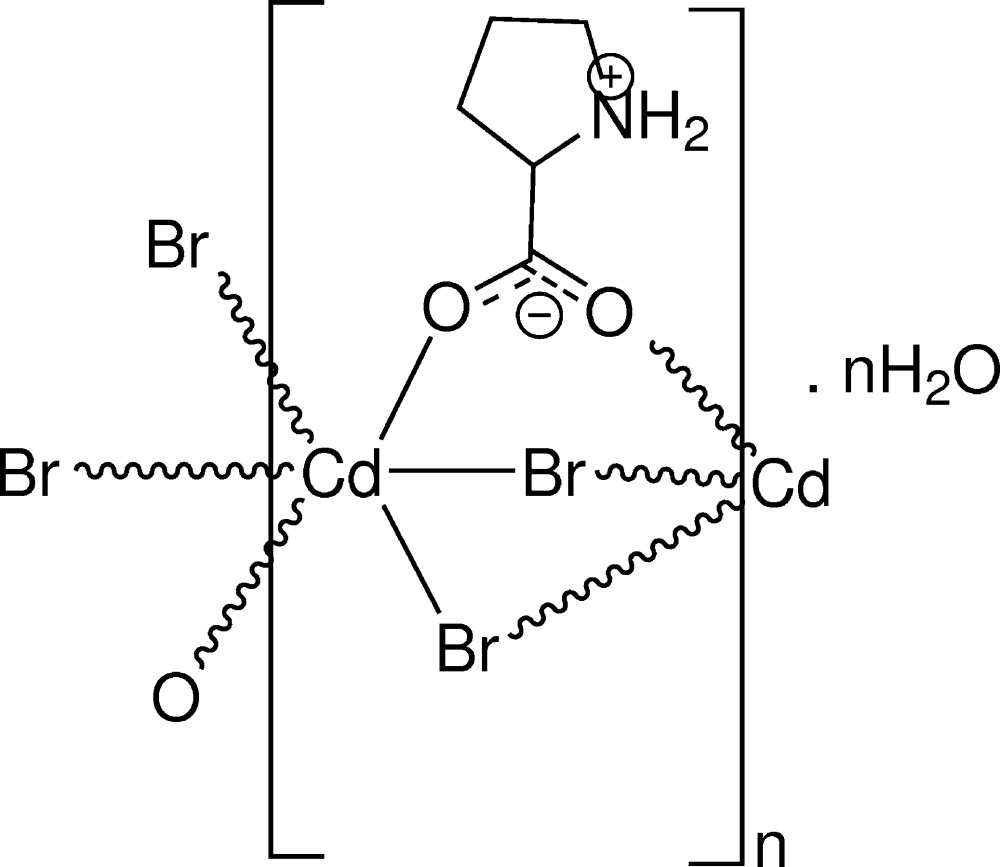


## Structural commentary   

**Figure 1:**
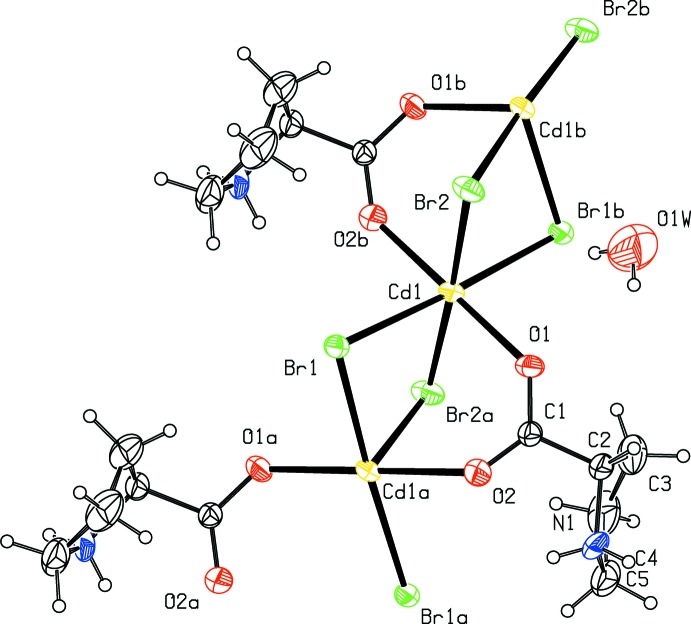
A portion of the crystal structure of the title complex, showing the atomic labeling. Displacement ellipsoids are drawn at the 30% probability level. [Symmetry codes: (*a*) 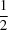 − *x*, −*y*, *z* − 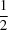; (*b*) 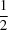 − *x*, −*y*, *z* + 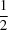.]

The asymmetric unit of the title complex (I) (Fig. 1 ▸) contains one Cd^II^ ion, one proline and two bromido ligands, and one water mol­ecule of crystallization. The title complex has a very similar structure to that of the chloride analogue (Yukawa *et al.*, 1983 ▸) and l-proline manganese dichloride monohydrate (Rzączyńska *et al.*, 1997 ▸; Lamberts & Englert, 2012 ▸). In (I), proline exists in a zwitterionic form, as evident from the bond lengths involving the carboxyl­ate atoms and the protonation of the ring N atom of the pyrrolidine fragment. The Cd^II^ ion is coordinated by four bromido ligands [Cd—Br = 2.7236 (13)–2.7737 (12) Å] and two carboxyl­ate oxygen atoms [Cd—O = 2.312 (8) and 2.318 (8) Å] of two proline ligands in a slightly distorted octa­hedral geometry. The title complex is extended as a polymeric chain which runs parallel to the *c* axis. Within one chain, adjacent Cd^II^ ions are separated by 3.727 (1) Å. The closest Cd⋯Cd distance between neighbouring polymeric chains is 8.579 (2) Å. The five endocyclic torsion angles of the pyrrolidine ring of the proline residue are N1—C2—C3—C4 = 31.8 (13)°, C2—C3—C4—C5 = −39.1 (15)°, C3—C4—C5—N1 = 29.9 (14)°, C2—N1—C5—C4 = −9.7 (12)° and C5—N1—C2—C3 = −13.1 (11)°. The pyrrolidine ring exhibits twisted conformation on the C3—C4 bond with a pseudo-rotation angle Δ = 249.3 (12)° and a maximum torsion angle ϕ_m_ = 38.5 (8)° (Rao *et al.*, 1981 ▸).

In (I), as observed in the chloride analogue (Yukawa *et al.*, 1983 ▸), there is an intra­molecular N1—H1*A*⋯O2 hydrogen bond between the amino group and the carboxyl­ate fragment.

## Supra­molecular features   

**Figure 2:**
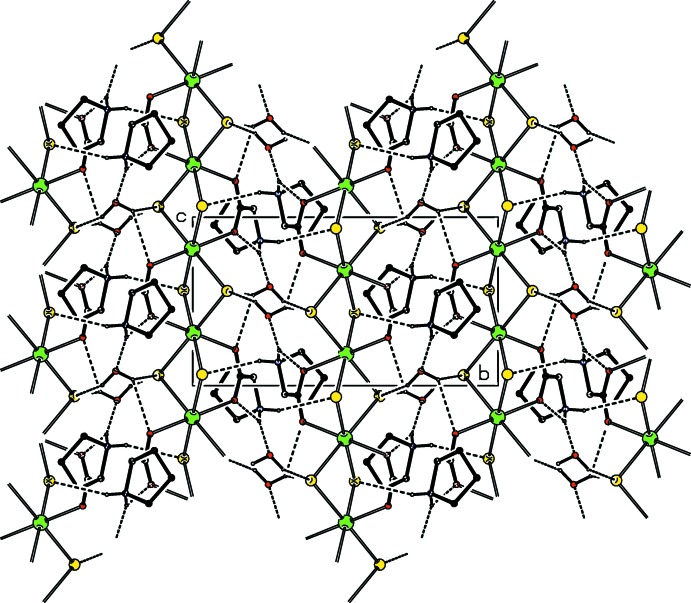
The crystal packing of (I) viewed along the *a* axis. Dashed lines denote inter­molecular hydrogen bonds. C-bound H atoms have been omitted for clarity.

**Figure 3:**
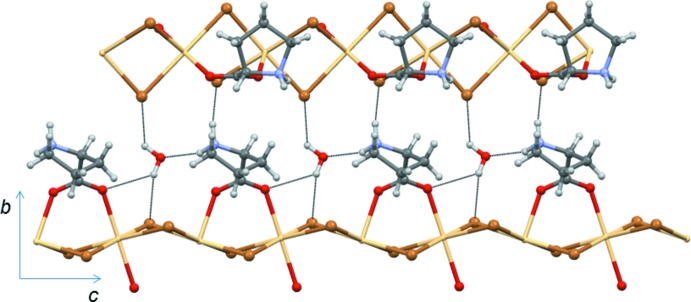
A portion of the crystal packing viewed along the *a* axis and showing hydrogen bonds (dashed lines) between two neighbouring polymeric chains.

The crystal structure of (I), is stabilized by inter­molecular N—H⋯O, N—H⋯Br, O—H⋯O and O—H⋯Br hydrogen bonds (Table 1 ▸, Figs. 2 ▸ and 3 ▸). The water mol­ecules serve as donors for the weak O—H⋯O and O—H⋯Br hydrogen bonds (Table 1 ▸) which link adjacent polymeric chains (Fig. 3 ▸), thus forming a three-dimensional structure.

## Database survey   

A search in the Cambridge Structural Database (Version 5.35, last update May 2014; Groom & Allen, 2014 ▸) for the structures with metal ions coordinated by one of the carboxyl­ate oxygen atoms of the proline moiety yielded 44 hits. Of these, two structures contain a cadmium metal ion, *viz. catena*-[di­chlorido-(4-hy­droxy-l-proline)cadmium] (refcode BOHVID; Yukawa *et al.*, 1982 ▸) and *catena*-[bis­(μ^2^-chlorido)(μ_2_-l-pro­line)cadmium monohydrate] (refcode BUXBUR; Yukawa *et al.*, 1983 ▸). The latter structure is isotypic with the title complex. Another compound, *catena*-[bis­(μ_2_-chlorido)(μ_2_-l-prolinato-κ^2^-*O*,*O*′)manganese(II) monohydrate], has been structurally determined three times and has similar cell parameters and the same space group as the title compound (refcode ROJQEM: Rzączyńska *et al.*, 1997 ▸; refcode ROJEQM01: Tilborg *et al.*, 2010 ▸; refcode ROJQEM02: Lamberts & Englert, 2012 ▸).

## Synthesis and crystallization   

To prepare the title compound, l-proline (Loba) and cadmium bromide tetra­hydrate (Loba) in an equimolar ratio were dissolved in double-distilled water. The obtained solution of the homogeneous mixture was evaporated at room temperature to afford the white crystalline title compound, which was then recrystallized by slow evaporation from an aqueous solution.

## Refinement   

Crystal data, data collection and structure refinement details are summarized in Table 2 ▸. As the title compound is isotypic with its chlorido analogue (Yukawa *et al.*, 1983 ▸), the atomic coordinates of the latter were used as starting values in the initial cycles of the refinement. The positions of water hydrogen atoms were calculated by method of Nardelli (1999 ▸). Further, the O—H and H1*W*⋯H2*W* distances of the water mol­ecules were restrained to 0.85 (2) and 1.38 (2) Å, respectively, using the DFIX option and included in the structure-factor calculations with *U*_iso_(H1*W*/H2*W*) = 1.1*U*_eq_(O1*W*). The remaining hydrogen atoms were placed in geometrically idealized positions (C—H = 0.97–0.98 Å and N—H = 0.89 Å) with *U*_iso_(H) = 1.2*U*_eq_(C/N) and were constrained to ride on their parent atoms. Reflections 110 and 020 were partially obscured by the beam stop and were omitted.

## Supplementary Material

Crystal structure: contains datablock(s) I. DOI: 10.1107/S2056989015001176/cv5483sup1.cif

Structure factors: contains datablock(s) I. DOI: 10.1107/S2056989015001176/cv5483Isup2.hkl

CCDC reference: 1044327

Additional supporting information:  crystallographic information; 3D view; checkCIF report

## Figures and Tables

**Table 1:** Hydrogen-bond geometry (, )

*D*H*A*	*D*H	H*A*	*D* *A*	*D*H*A*
N1H1*A*O2	0.89	2.16	2.626(12)	112
O1*W*H2*W*O1	0.84(17)	2.6(2)	3.175(19)	132
O1*W*H2*W*Br2	0.84(17)	2.8(3)	3.311(19)	123
N1H1*A*O1*W* ^i^	0.89	2.05	2.90(2)	159
N1H1*B*Br1^ii^	0.89	2.69	3.416(11)	140
O1*W*H1*W*Br2^iii^	0.88(16)	2.7(3)	3.197(19)	116

Symmetry codes: (i) 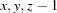; (ii) 
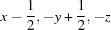
; (iii) 
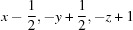
.

**Table 2:** Experimental details

Crystal data	
Chemical formula	[CdBr_2_(C_5_H_9_NO_2_)]H_2_O
*M*_r_	405.37
Crystal system, space group	Orthorhombic, *P*2_1_2_1_2_1_
Temperature (K)	296
*a*, *b*, *c* ()	10.1891(8), 13.4961(11), 7.4491(5)
*V* (^3^)	1024.35(13)
*Z*	4
Radiation type	Mo *K*
(mm^1^)	9.90
Crystal size (mm)	0.35 0.30 0.30

Data collection	
Diffractometer	Bruker *SMART* CCD area detector
Absorption correction	Multi-scan (*SADABS*; Bruker, 2008 ▸)
*T*_min_, *T*_max_	0.129, 0.155
No. of measured, independent and observed [*I* > 2(*I*)] reflections	8264, 2481, 1964
*R*_int_	0.068
(sin /)_max_ (^1^)	0.666

Refinement	
*R*[*F* ^2^ > 2(*F* ^2^)], *wR*(*F* ^2^), *S*	0.041, 0.089, 1.06
No. of reflections	2481
No. of parameters	115
No. of restraints	3
H-atom treatment	H atoms treated by a mixture of independent and constrained refinement
_max_, _min_ (e ^3^)	1.02, 1.07
Absolute structure	Flack *x* determined using 705 quotients [(*I* ^+^)(*I* )]/[(*I* ^+^)+(*I* )] (Parsons *et al.*, 2013 ▸)
Absolute structure parameter	0.035(15)

Computer programs: *APEX2*, *SAINT* and *XPREP* (Bruker, 2008 ▸), *SHELXL2014*/6 (Sheldrick, 2015 ▸), *PLATON* (Spek, 2009 ▸) and *Mercury* (Macrae *et al.*, 2008 ▸).

## supplementary crystallographic information

### Crystal data

**Table:**

[CdBr_2_(C_5_H_9_NO_2_)]·H_2_O	*D*_x_ = 2.629 Mg m^−^^3^
*M**_r_* = 405.37	Mo *K*α radiation, λ = 0.71073 Å
Orthorhombic, *P*2_1_2_1_2_1_	Cell parameters from 4066 reflections
*a* = 10.1891 (8) Å	θ = 5.0–55.2°
*b* = 13.4961 (11) Å	µ = 9.90 mm^−^^1^
*c* = 7.4491 (5) Å	*T* = 296 K
*V* = 1024.35 (13) Å^3^	Block, colourless
*Z* = 4	0.35 × 0.30 × 0.30 mm
*F*(000) = 760	

### Data collection

**Table:**

Bruker SMART CCD area detector diffractometer	1964 reflections with *I* > 2σ(*I*)
Radiation source: fine-focus sealed tube	*R*_int_ = 0.068
ω and φ scan	θ_max_ = 28.2°, θ_min_ = 3.1°
Absorption correction: multi-scan (*SADABS*; Bruker, 2008)	*h* = −13→13
*T*_min_ = 0.129, *T*_max_ = 0.155	*k* = −17→14
8264 measured reflections	*l* = −9→6
2481 independent reflections	

### Refinement

**Table:**

Refinement on *F*^2^	Hydrogen site location: mixed
Least-squares matrix: full	H atoms treated by a mixture of independent and constrained refinement
*R*[*F*^2^ > 2σ(*F*^2^)] = 0.041	*w* = 1/[σ^2^(*F*_o_^2^) + (0.0243*P*)^2^ + 1.4185*P*] where *P* = (*F*_o_^2^ + 2*F*_c_^2^)/3
*wR*(*F*^2^) = 0.089	(Δ/σ)_max_ < 0.001
*S* = 1.06	Δρ_max_ = 1.02 e Å^−^^3^
2481 reflections	Δρ_min_ = −1.07 e Å^−^^3^
115 parameters	Absolute structure: Flack *x* determined using 705 quotients [(*I*^+^)-(*I*^-^)]/[(*I*^+^)+(*I*^-^)] (Parsons *et al.*, 2013)
3 restraints	Absolute structure parameter: 0.035 (15)

### Special details

**Table:**

Geometry. All e.s.d.'s (except the e.s.d. in the dihedral angle between two l.s. planes) are estimated using the full covariance matrix. The cell e.s.d.'s are taken into account individually in the estimation of e.s.d.'s in distances, angles and torsion angles; correlations between e.s.d.'s in cell parameters are only used when they are defined by crystal symmetry. An approximate (isotropic) treatment of cell e.s.d.'s is used for estimating e.s.d.'s involving l.s. planes.

### Fractional atomic coordinates and isotropic or equivalent isotropic displacement parameters (Å^2^)

**Table:**

	*x*	*y*	*z*	*U*_iso_*/*U*_eq_	
Cd1	0.24415 (7)	0.00192 (7)	0.31349 (9)	0.0425 (2)	
Br1	0.44442 (8)	0.03071 (8)	0.06673 (14)	0.0450 (3)	
Br2	0.37743 (10)	0.11262 (9)	0.56256 (15)	0.0537 (3)	
O1	0.1309 (8)	0.1397 (6)	0.2136 (9)	0.057 (2)	
O2	0.1420 (7)	0.1362 (6)	−0.0865 (9)	0.056 (2)	
N1	−0.0870 (10)	0.2205 (8)	−0.1393 (11)	0.062 (3)	
H1A	−0.0168	0.2171	−0.2100	0.075*	
H1B	−0.1202	0.2813	−0.1471	0.075*	
C1	0.0861 (9)	0.1560 (7)	0.0564 (15)	0.039 (2)	
C2	−0.0488 (10)	0.1988 (8)	0.0510 (15)	0.053 (3)	
H2	−0.0524	0.2596	0.1229	0.064*	
C3	−0.1523 (12)	0.1260 (13)	0.115 (2)	0.084 (5)	
H3A	−0.1172	0.0826	0.2066	0.100*	
H3B	−0.2279	0.1607	0.1627	0.100*	
C4	−0.1878 (13)	0.0697 (13)	−0.047 (2)	0.094 (5)	
H4A	−0.2733	0.0392	−0.0326	0.113*	
H4B	−0.1236	0.0181	−0.0701	0.113*	
C5	−0.1899 (14)	0.1441 (12)	−0.200 (2)	0.086 (5)	
H5A	−0.2758	0.1743	−0.2126	0.103*	
H5B	−0.1651	0.1134	−0.3127	0.103*	
O1W	0.111 (2)	0.2521 (17)	0.587 (2)	0.183 (8)	
H1W	0.11 (3)	0.296 (11)	0.50 (2)	0.201*	
H2W	0.13 (3)	0.197 (8)	0.54 (3)	0.201*	

### Atomic displacement parameters (Å^2^)

**Table:**

	*U*^11^	*U*^22^	*U*^33^	*U*^12^	*U*^13^	*U*^23^
Cd1	0.0453 (4)	0.0579 (4)	0.0243 (3)	0.0069 (4)	−0.0005 (2)	0.0045 (3)
Br1	0.0347 (4)	0.0679 (7)	0.0323 (4)	0.0033 (5)	−0.0007 (4)	−0.0001 (5)
Br2	0.0597 (6)	0.0687 (7)	0.0327 (5)	−0.0117 (6)	0.0013 (5)	−0.0056 (6)
O1	0.074 (5)	0.066 (5)	0.032 (4)	0.025 (4)	−0.011 (3)	−0.005 (4)
O2	0.059 (5)	0.068 (5)	0.043 (4)	0.016 (4)	0.005 (4)	0.007 (4)
N1	0.063 (6)	0.066 (7)	0.058 (6)	0.037 (6)	−0.015 (5)	−0.001 (5)
C1	0.040 (5)	0.039 (5)	0.039 (5)	0.005 (4)	−0.002 (5)	−0.003 (5)
C2	0.053 (6)	0.060 (7)	0.046 (5)	0.024 (6)	−0.009 (6)	−0.010 (6)
C3	0.043 (7)	0.113 (13)	0.095 (10)	0.005 (8)	0.018 (6)	0.008 (10)
C4	0.042 (6)	0.110 (12)	0.130 (13)	−0.008 (8)	0.006 (9)	−0.021 (13)
C5	0.075 (9)	0.090 (11)	0.091 (10)	0.040 (9)	−0.024 (8)	−0.037 (9)
O1W	0.178 (16)	0.22 (2)	0.153 (13)	0.061 (18)	0.007 (14)	0.061 (17)

### Geometric parameters (Å, º)

**Table:**

Cd1—O1	2.312 (8)	N1—H1B	0.8900
Cd1—O2^i^	2.318 (8)	C1—C2	1.491 (13)
Cd1—Br2^ii^	2.7236 (13)	C2—C3	1.517 (19)
Cd1—Br1^i^	2.7285 (11)	C2—H2	0.9800
Cd1—Br2	2.7421 (13)	C3—C4	1.47 (2)
Cd1—Br1	2.7737 (12)	C3—H3A	0.9700
Br1—Cd1^ii^	2.7285 (11)	C3—H3B	0.9700
Br2—Cd1^i^	2.7236 (13)	C4—C5	1.52 (2)
O1—C1	1.276 (12)	C4—H4A	0.9700
O2—C1	1.237 (12)	C4—H4B	0.9700
O2—Cd1^ii^	2.318 (8)	C5—H5A	0.9700
N1—C2	1.499 (13)	C5—H5B	0.9700
N1—C5	1.537 (17)	O1W—H1W	0.87 (3)
N1—H1A	0.8900	O1W—H2W	0.87 (3)
			
O1—Cd1—O2^i^	179.9 (3)	O1—C1—C2	114.9 (9)
O1—Cd1—Br2^ii^	90.50 (19)	C1—C2—N1	109.9 (9)
O2^i^—Cd1—Br2^ii^	89.53 (19)	C1—C2—C3	112.4 (10)
O1—Cd1—Br1^i^	90.0 (2)	N1—C2—C3	103.9 (10)
O2^i^—Cd1—Br1^i^	90.03 (19)	C1—C2—H2	110.1
Br2^ii^—Cd1—Br1^i^	93.59 (4)	N1—C2—H2	110.1
O1—Cd1—Br2	91.52 (19)	C3—C2—H2	110.1
O2^i^—Cd1—Br2	88.44 (19)	C4—C3—C2	104.5 (11)
Br2^ii^—Cd1—Br2	177.29 (3)	C4—C3—H3A	110.9
Br1^i^—Cd1—Br2	88.22 (3)	C2—C3—H3A	110.9
O1—Cd1—Br1	92.4 (2)	C4—C3—H3B	110.9
O2^i^—Cd1—Br1	87.56 (19)	C2—C3—H3B	110.9
Br2^ii^—Cd1—Br1	87.67 (4)	H3A—C3—H3B	108.9
Br1^i^—Cd1—Br1	177.27 (4)	C3—C4—C5	106.0 (12)
Br2—Cd1—Br1	90.44 (4)	C3—C4—H4A	110.5
Cd1^ii^—Br1—Cd1	85.27 (3)	C5—C4—H4A	110.5
Cd1^i^—Br2—Cd1	85.98 (3)	C3—C4—H4B	110.5
C1—O1—Cd1	127.7 (6)	C5—C4—H4B	110.5
C1—O2—Cd1^ii^	132.9 (7)	H4A—C4—H4B	108.7
C2—N1—C5	108.9 (10)	C4—C5—N1	102.3 (10)
C2—N1—H1A	109.9	C4—C5—H5A	111.3
C5—N1—H1A	109.9	N1—C5—H5A	111.3
C2—N1—H1B	109.9	C4—C5—H5B	111.3
C5—N1—H1B	109.9	N1—C5—H5B	111.3
H1A—N1—H1B	108.3	H5A—C5—H5B	109.2
O2—C1—O1	126.0 (8)	H1W—O1W—H2W	106 (4)
O2—C1—C2	119.0 (10)		
			
Cd1^ii^—O2—C1—O1	44.5 (15)	C5—N1—C2—C1	107.4 (11)
Cd1^ii^—O2—C1—C2	−132.7 (9)	C5—N1—C2—C3	−13.1 (11)
Cd1—O1—C1—O2	−40.4 (15)	C1—C2—C3—C4	−87.0 (14)
Cd1—O1—C1—C2	136.8 (8)	N1—C2—C3—C4	31.8 (13)
O2—C1—C2—N1	−6.1 (15)	C2—C3—C4—C5	−39.1 (15)
O1—C1—C2—N1	176.4 (9)	C3—C4—C5—N1	29.9 (14)
O2—C1—C2—C3	109.1 (12)	C2—N1—C5—C4	−9.7 (12)
O1—C1—C2—C3	−68.3 (13)		

Symmetry codes: (i) −*x*+1/2, −*y*, *z*+1/2; (ii) −*x*+1/2, −*y*, *z*−1/2.

### Hydrogen-bond geometry (Å, º)

**Table:**

*D*—H···*A*	*D*—H	H···*A*	*D*···*A*	*D*—H···*A*
N1—H1*A*···O2	0.89	2.16	2.626 (12)	112
O1*W*—H2*W*···O1	0.84 (17)	2.6 (2)	3.175 (19)	132
O1*W*—H2*W*···Br2	0.84 (17)	2.8 (3)	3.311 (19)	123
N1—H1*A*···O1*W*^iii^	0.89	2.05	2.90 (2)	159
N1—H1*B*···Br1^iv^	0.89	2.69	3.416 (11)	140
O1*W*—H1*W*···Br2^v^	0.88 (16)	2.7 (3)	3.197 (19)	116

Symmetry codes: (iii) *x*, *y*, *z*−1; (iv) *x*−1/2, −*y*+1/2, −*z*; (v) *x*−1/2, −*y*+1/2, −*z*+1.
